# High Precision Detection Method for Delamination Defects in Carbon Fiber Composite Laminates Based on Ultrasonic Technique and Signal Correlation Algorithm

**DOI:** 10.3390/ma13173840

**Published:** 2020-08-31

**Authors:** Mengyuan Ma, Hongyi Cao, Mingshun Jiang, Lin Sun, Lei Zhang, Faye Zhang, Qingmei Sui, Aiqin Tian, Jianying Liang, Lei Jia

**Affiliations:** 1 School of Control Science and Engineering, Shandong University, Ji’nan 250061, China; chinamamengyuan@163.com (M.M.); chy_sdu@163.com (H.C.); drleizhang@sdu.edu.cn (L.Z.); zhangfaye@sdu.edu.cn (F.Z.); qmsui@sdu.edu.cn (Q.S.); jialei@sdu.edu.cn (L.J.); 2 Zhongche Qingdao Sifang Locomotive and Rolling Stock Co., Ltd, Qingdao 266111, China; sfsunlin@aliyun.com (L.S.); sftianaiqin@sina.com (A.T.); sfliangjianying@sina.com (J.L.)

**Keywords:** non-destructive testing, composites, ultrasonic testing, delamination, signal correlation, defect imaging

## Abstract

This paper presents a method based on signal correlation to detect delamination defects of widely used carbon fiber reinforced plastic with high precision and a convenient process. The objective of it consists in distinguishing defect and non-defect signals and presenting the depth and size of defects by image. A necessary reference signal is generated from the non-defect area by using autocorrelation theory firstly. Through the correlation calculation results, the defect signal and non-defect signal are distinguished by using Euclidean distance. In order to get more accurate time-of-flight, cubic spline interpolation is introduced. In practical automatic ultrasonic A-scan signal processing, signal correlation provide a new way to avoid problems such as signal peak tracking and complex gate setting. Finally, the detection results of a carbon fiber laminate with artificial delamination through ultrasonic phased array C-scan acquired from Olympus OmniScan MX2 and this proposed algorithm are compared, which showing that this proposed algorithm performs well in defect shape presentation and location calculation. The experiment shows that the defect size error is less than 4%, the depth error less than 3%. Compared with ultrasonic C-scan method, this proposed method needs less inspector’s prior-knowledge, which can lead to advantages in automatic ultrasonic testing.

## 1. Introduction

The carbon fiber reinforced plastic (CFRP) has been widely used in aerospace [1], military [2], automotive [3] and other fields, because of its good performance in weight and strength. Due to its multilayer structure and special manufacturing process, defects which reduce its mechanical properties and lead to structural problems, are prone to appear in the manufacturing process, such as delamination [4,5], inclusion [6,7].

Delamination is one of the most important defects of composite materials, and it is also an important factor causing composites failure. Many researchers focus on the detection, analysis and prediction of delamination defects [5]. In work by Garcia et al. [8], a new approach to predict the delamination caused by impacts in composite structures was proposed based on the electric responses of triboelectric sensor, and the delamination failures of glass fiber epoxy composite laminates were analyzed. The delamination defects continue to expand due to stress and impact damage [9] during service, which greatly reduces the strength of the structural parts, resulting in overall structural failure or other catastrophic consequences. Therefore, the detection of defects which can distinguish the non-defect and defective CFRP, is extremely important for the wide application of CFRP. In order to more accurately evaluate the performance of the composite material, the shape, size and location of the delamination defect need to be obtained, in which fields ultrasonic testing performs well. The ultrasonic testing has been one of the mostly extensively used nondestructive testing technologies [10,11,12].

The ultrasonic testing is based on the detection and the feature extraction of the ultrasonic waves reflected by defects. From the ultrasonic acquisition device, we can only get ultrasonic A-scan signals. In order to identify the defect from the ultrasonic echo signal, there are some methods should be used to process ultrasonic signals. There are many existing methods of ultrasonic body wave and guided wave signal processing [13,14], such as split spectrum processing (SSP) [15,16], wavelet transform [17,18], Hilbert–Huang transform [19] and S-transform [20,21].

When using SSP, selecting parameters such as filter type, center frequency, and bandwidth is a tricky task. Rodríguez et al. [22] researched on a new split spectrum processing method which combined Frequency Multiplication (FM) and equalized and equally spaced variable bandwidth filter bank (E-ESVB). Bouden et al. [23] proposed an Adaptive Split Spectrum Processing technique (A-SSP) based on Empirical Mode Decomposition (EMD). Although their work improves the parameter selection of SSP through intelligent algorithms, there is no uniform standard for the parameters that need to be changed when detecting different objects. The main application of wavelet transform in ultrasonic testing is denoising. Praveen et al. [24] used higher order wavelets to enhance ultrasonic signal quality. In work by Mohammadkhani et al. [25], the strongest echo which can be found from ultrasonic echo signal by wavelet transform, is combined with echo-fit search methodology to locate all effective echoes through multiple iterations. In work by Luo et al. [26], a new wavelet thresholding function based on soft and hard thresholding functions was proposed to denoise the ultrasonic signal and the SNR reached 17.2634. However, in the wavelet transform, the choice of wavelet function and threshold greatly affects the result, which leads to limitations in automated ultrasonic testing. Hilbert–Huang transform is a effective tool for ultrasonic signal analysis [27]. Sharma et al. [28] combined the Ensemble Empirical Mode Decomposition (EEMD) processing and signal minimisation algorithm to reconstruct ultrasonic signals. Ali et al. [29] used Hilbert–Huang transform to classify ultrasonic signals in terms of different levels of thermal fatigue, in order to detect mild steel welded areas. Shi et al. [30] applied Hilbert–Huang transform to non-destructive sound wave testing of full-length bonded rock bolts. S-transform combines the time-frequency representation of the Gabor transform and multi-resolution feature of wavelet transform [31]. Benamma et al. [32] proposed a new modified S-transform based on thresholding technique in order to get a better time frequency resolution. Xu [33] used the frequency energy spectrum with S transform to identify defects in the concrete structure. Zhu et al. [34] proposed a denoising algorithm based on the generalized S transform and singular value decomposition (SVD) to denoise echo signals of ultrasonic pulse-echo testing.

Although the above-mentioned algorithms can extract the characteristics of the defect well and achieve better detection results compared with classical ultrasonic C-scan method, in the process of using these algorithms, some parameters such as thresholds need to be set. Some algorithms are complex and take a long time to process ultrasonic signals. These algorithms are difficult to use for automated ultrasonic testing. As a result, classical ultrasonic C-scan is still the main method of automated ultrasonic testing.

In order to find an accurate and efficient automated ultrasonic signal processing algorithm, signal correlation technology is introduced. By using signal correlation, some problem in classical ultrasonic C-scan testing such as signal peak tracking and complex gate setting can be avoided. As a classical signal processing method, signal correlation processing has been also applied to the processing of ultrasonic signals [35,36,37]. In the work by Kawamura et al. [38], in order to investigate the depth of boulder penetration, autocorrelation was used to identify the time tag of the reflection wave in acoustic emission (AE). In the work by Liang et al. [39], a maximum fractional cross-correlation spectrum (FCCS) parameter estimation method was proposed to estimate the time of arrival (TOA) of the ultrasonic echoes. Luppescu et al. [40] increased both the signal-to-noise ratio and time-resolution of ultrasonic guided wave measurements by autocorrelation. However, in the ultrasonic pulse echo detection, the correlation was rarely used. Li et al. [41] used signal correlation to classify defect and non-defect signals. However, in nondestructive testing, it is not only necessary to distinguish between defects and non-defects, but also to analyze quantitatively the shape, size and depth of the defects. Further research is needed.

In this paper, the ultrasonic signals are processed based on signal correlation. First, the acquired A-scan signal is autocorrelated in order to avoid some problems and complex operations in classical ultrasonic C-scan such as the complex gate settings, and generate reference autocorrelation from the non-defect area. Second, Euclidean distances are used to distinguish between defective and non-defective signals. For defective signals, time of flight (TOF) is calculated using signal correlation and cubic spline interpolation. Finally, defect images are generated, similar to C-scan images. Through the experimental detection of a laminate with artificial delamination defects and compared with the result of ultrasonic phased array equipment (OmniScan MX2, Olympus, Tokyo, Japan), it can be concluded that the proposed algorithm presents the shape, size and location of the defects well, with defect size error less than 4% and the depth error less than 3%.

## 2. Proposed Method Based on Signal Correlation

### 2.1. Classical Ultrasonic C-Scan Method

Ultrasonic C-scan imaging technology has been widely used in many nondestructive testing fields for defect detection. The original signal acquired from the ultrasound acquisition card is a set of A-scan signals, of which each signal corresponds to a detection position and contains the defect information. Ultrasound C-scan uses the amplitude or TOF (time of flight) in the A-scan signal to generate a two-dimensional image [42,43]. In this process, the choice of gate location and threshold is very important, which can greatly affect the accuracy of defect detection and ultrasonic C-scan imaging. However, setting appropriate gate position and threshold value has higher requirements for inspectors and requires prior knowledge.

**Figure 1:**
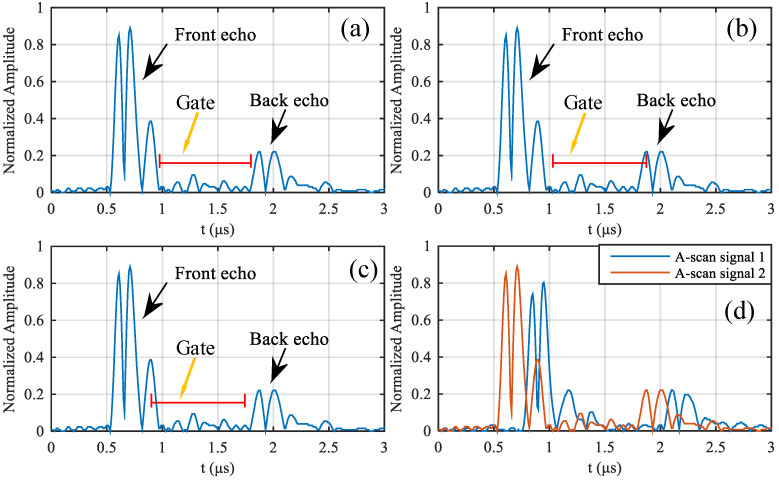
Ultrasonic A-scan signals with (**a**) proper gate, (**b**) gate too right and (**c**) gate too left. (**d**) Ultrasound A-scan signals at different positions in the same scan, which reflects the time shift of the signals. (All signals are obtained by full-wave rectification from ultrasonic A-scan signals.).

In classical ultrasonic C-scan, the gate contains three variables, namely the starting point, the length and the threshold. The starting point and length determine the location to be tested. In the gate range, if there is an echo greater than the threshold, it is judged that there is a defect here. For example, when detecting internal defects in the laminate, the gate is usually placed between the front echo (reflected on the upper surface of the laminate) and the back echo (reflected on the bottom surface of the laminate), as shown in Figure 1a. If set too right or too left, the gate will detect the back echo or front echo as defect as shown in Figure 1b,c. Therefore, in order to reduce the impact of front echo and back echo, the length of the gate is usually set to be less than the depth of the laminate. As a result, the classic ultrasonic C-scan method is difficult to detect near-surface defects, especially its depth calculation is poor.

In addition, because of the slight change in the distance between the ultrasonic transducer and the laminate which can lead to signal time shift, the position of the gate needs to be adjusted in real time, as shown in Figure 1d. In the traditional ultrasonic C-scan, the above problem is solved by tracking the front echo peak and keeping the relative distance between the gate and the front echo peak constant. Therefore, signal peak tracking is necessary. However, due to the influence of noise, the signal tracking often has errors, so that the position of the gate cannot be in the correct position.

### 2.2. A-Scan Signal Correlation Algorithm

In this paper, we consider that the various echoes in the ultrasonic A-scan signals are uncorrelated and independent variables in the time domain. Therefore, the autocorrelation can be used to process the ultrasound A-scan signal. autocorrelation can be considered as the cross-correlation of a signal with itself.

For the first-order autocorrelation, the shift is one time unit. Assume that a time series $x_{i}\left(i=1,\;2,\;\cdots ,\;N\right)$ is an A-scan signal, where *N* is the number of points of the signal, and $x_{i}$ as the signal amplitude at time *i*. The first-order autocorrelation coefficient is given by

(1) $$R_{1}=\frac{\sum_{i=1}^{N-1}\left(x_{i}-\bar{x}\right)\left(x_{i+1}-\bar{x}\right)}{\sum_{i=1}^{n}{\left(x_{i}-\bar{x}\right)}^{2}}$$

where $\bar{x}$ is the mean. The k order autocorrelation coefficient is given by

(2) $$R_{k}=\frac{\sum_{i=k}^{N-k}\left(x_{i}-\bar{x}\right)\left(x_{i+k}-\bar{x}\right)}{\sum_{i=1}^{n}{\left(x_{i}-\bar{x}\right)}^{2}}$$

### 2.3. Overview of The Method

The overview of the proposed method based on signal correlation is shown as follows.

- Step 1: Acquire standard A-scan signals from non-defect area. In order to reduce the influence of random errors, the number of signals should be greater than 10.
- Step 2: Calculate autocorrelation from above standard A-scan signals and generate reference autocorrelation, which is detailed in Section 2.4.
- Step 3: Ultrasonic scan of all areas of the object to obtain A-scan signals, during which the parameters such as the distance between the ultrasonic transducer and the object to be measured, the ultrasonic excitation pulse voltage, the ultrasonic echo signal sampling rate, and the signal gain should be set to be consistent with Step 1.
- Step 4: Calculate autocorrelation for all A-scan signals, and classify signals from defect and non-defect, which is detailed in Section 2.5.
- Step 5: Generate the defect image by Euclidean distance rather than the amplitude of the defect echo used in traditional ultrasonic C-scan. To improve visibility, we use pseudo-color coding to convert gray images into color images.
- Step 6: Calculate the defect depth of the A-scan signals of the classified defect area, which is detailed in Section 2.6.

### 2.4. Generate Reference Signal

Reference signal should have the characteristics of non-defect signals. As a result, it can be calculated from signals of non-defect area. In order to reduce the influence of noise and random error, a set of signals from non-defect area should be collected to generate the reference signal. Assume that the number of signals collected is *n*. Mean value is used for generating reference signal, shown as follows.

(3) $$ref\left(t\right)=\frac{1}{n}\sum_{i=1}^{n}corr\left(t,i\right)$$

where ref(t) is the reference autocorrelation, corr(t,i) is autocorrelation of the *i*th signal.

### 2.5. Classification of Defective and Non-Defective Signals

In order to classify the characteristics of defective and non-defective signals, Euclidean distance is introduced. *X* and *Y* are two vectors on an n-dimensional Euclidean space $R^{n}$. The Euclidean distance between $X=(x_{1},\;x_{2},\;\cdots ,\;x_{n})$ and $Y=(y_{1},\;y_{2},\;\cdots ,\;y_{n})$ is given by

(4) $$dist\left(X,Y\right)=\sqrt{\sum_{i=1}^{n}{\left(x_{i}-y_{i}\right)}^{2}}$$

Euclidean distance between autocorrelation of signals to be classified and reference autocorrelation is utilized as basis to classify defective and non-defective signals. The Euclidean distance is given by

(5) $$dist\left(corr,ref\right)=\sqrt{\sum_{i=1}^{n}{\left(corr_{i}-ref_{i}\right)}^{2}}$$

where $corr_{i}=corr\left(t_{i}\right),\left(i=1,\;2,\;3,\;\cdots ,\;n\right)$ is the autocorrelation sequence of signals to be classified and $ref_{i}=ref\left(t_{i}\right),\left(i=1,\;2,\;3,\;\cdots ,\;n\right)$ is the reference autocorrelation sequence calculated by Function 3. Because the reference autocorrelation is calculated by A-scan signals without defect, Euclidean distance is larger when calculated by defective signals. We can set a threshold to classify defective and non-defective signals. The threshold can be calculated by a set of *m* non-defective signals, as follows.

(6) $$threshold=k\frac{1}{m}\sum_{j=1}^{m}\left(dist_{j}\right)$$

where $dist_{j}$ is the *j*th Euclidean distance between autocorrelation of non-defective signal and reference autocorrelation calculated by Fuction 5, *k* is the variable coefficient. According to experience, *k* can be set to 3.

### 2.6. Calculation of Defect Depth

Assuming that the speed of sound in test object is constant, the defect depth can be calculated from time-of-flight. In this paper, a new algorithm for time-of-flight is proposed based on signal correlation. Compared with Hilbert–Huang transform, if signal correlation used alone, the accuracy of result is poor. Therefore, cubic spline interpolation algorithm is introduced.

**Figure 2:**
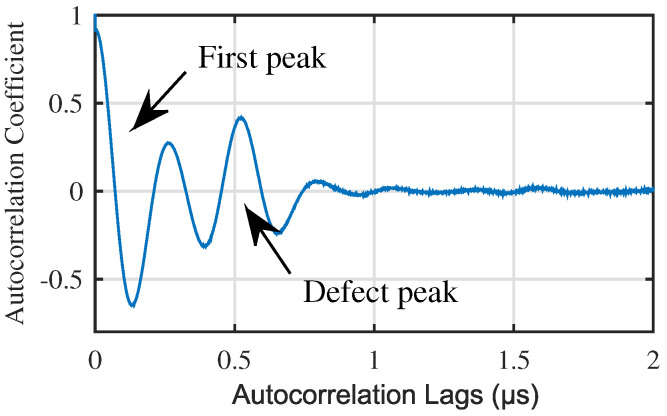
Impact of the first peak of autocorrelation.

**Figure 3:**
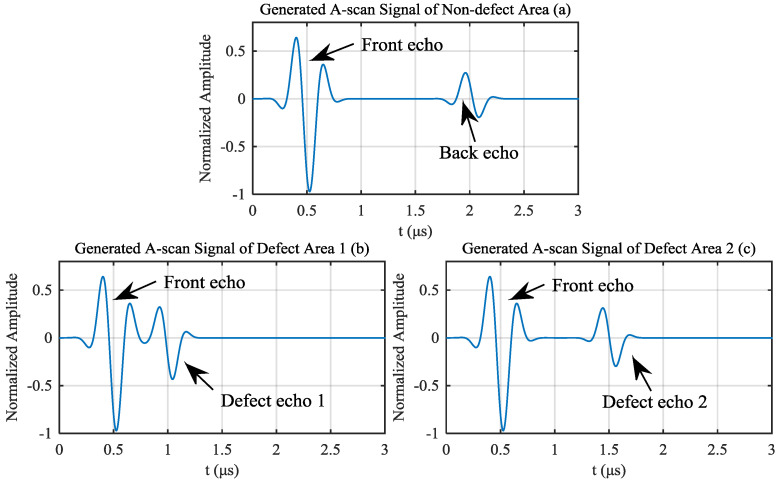
Generated signals of (**a**) non-defect area; (**b**) defect area 1 and (**c**) defect area 2.

First, we should reduce the impact of the first peak of the autocorrelation, as shown in Figure 2, which is the autocorrelation of a generated noisy ultrasonic A-scan signal for near surface defect. The signal generation method is introduced in Section 3. In Figure 2, the first peak is owing to zero lag of the autocorrelation, and the defect peak is owing to the correlation between front echo and defect echo (front echo and defect echo as shown in Figure 3b). When near surface defect, the cross-aliasing of defect echo and front echo results in the error of defect location. Similarity, the small distance between first peak and defect peak of the autocorrelation affects the following extraction of defect peak. In short, it is necessary to reduce the influence of the first peak of autocorrelation.

The specific algorithm as follows:

- Step 1: Intercept the first peak of the reference autocorrelation, record as $S_{FP}\left(t\right)$.
- Step 2: Calculate the defect peak by the follow function.
  (7) $$S_{D}\left(t\right)=corr\left(t\right)-S_{FP}\left(t\right)$$
  where $S_{D}\left(t\right)$ is the defect peak, corr(t) is the autocorrelation of defect signal, $S_{FP}\left(t\right)$ is the first peak of the reference autocorrelation.
- Step 3: Use median filter to smooth the curve of defect peak, and get $S_{D}^{*}\left(t\right)$.
- Step 4: Find the peaks of $\left|S_{D}^{*}\left(t\right)\right|$, as $\left(t_{i},\left|S_{D}^{*}\left(t_{i}\right)\right|\right)$, where *i* is the order number of peaks. Use cubic spline interpolation to interpolate these peaks, record as $S_{C}\left(t\right)$. Set the interval as one tenth of the original signal interval.
- Step 5: Find the maximum value of $S_{C}\left(t\right)$, record as $S_{CMax}=S_{C}\left(t_{m}\right)$. The $t_{m}$ corresponding to the maximum value is the required sound path. Finally, the defect depth can be calculated from $t_{m}$.

### 2.7. Advantages of Proposed Algorithm over Traditional Ultrasonic C-Scan Method

Traditional ultrasonic C-scan method needs signal peak tracking and complex gate setting, which is illustrated in Section 2.1. The proposed algorithm can avoid these two problems. On one hand, without using gate, the proposed algorithm can classify defective and non-defective signals by Euclidean distance between autocorrelation of reference signal and signals to be classified. Therefore, gate setting is avoided, and operator can conduct ultrasonic testing by proposed algorithm with less prior knowledge. On the other hand, the effective signal in ultrasound A scan starts from the front echo, and its starting position is not fixed due to the time shift of the signal. However, after the autocorrelation calculation, the effective domain of the autocorrelation is fixed and from zero. Therefore, tracking of signal peaks is avoided. What is more, when calculating the defect depth, the proposed method uses the absolute position of the defect peak instead of using the relative distance between front echo and defect echo as in traditional ultrasonic C-scan, which makes the calculation more convenient.

## 3. Simulation Based on Artificial Echo Signals

In this section, a set of non-defect signals and delamination defect signals were generated manually. The Gaussian echo model of ultrasonic signal was used, which is shown as follows [44].

(8) $$s\left(t,\theta \right)=\beta e^{-\alpha {\left(t-\tau \right)}^{2}}cos\left(2\pi f_{c}\left(t-\tau \right)+\phi \right)$$

where parameter vector $\theta =\left[\alpha ,\;\tau ,\;f_{c},\;\phi ,\;\beta \right]$ is the characteristic parameter of the echo, α is related to bandwidth, τ is arrival time, $f_{c}$ is center frequency, ϕ is the phase and β is the amplitude coefficient.

**Figure 4:**
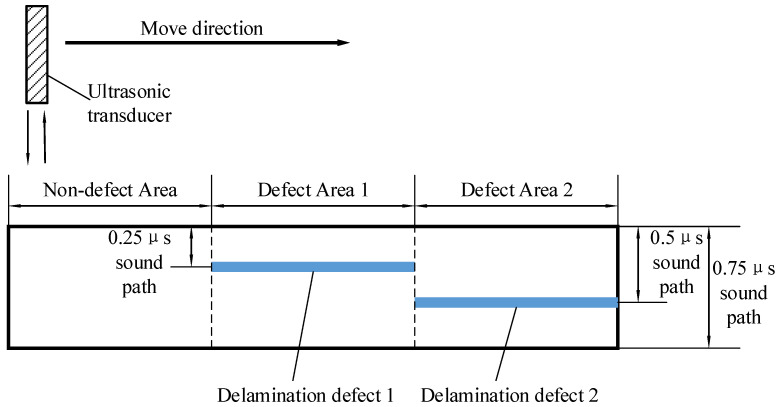
Assumed detecting condition and delamination defect location.

Assume that the ultrasonic testing condition is shown in Figure 4, and the object to be test has three different areas, non-defect area, defect area 1 and defect area 2. The thickness of the object is 0.75 μs sound path, the depth of defect 1 is 0.25 μs sound path and the depth of defect 2 is 0.5 μs sound path. The generated non-defect signals and defect signals are shown as Figure 3. Assume that ultrasonic echo satisfies the Gaussian echo model (8) and no interference from structure noise under ideal conditions. The A-scan signal of non-defect area can be generated by adding front echo and back echo, as shown in Figure 3a, where front echo represents the reflection of ultrasonic waves on the upper surface of the composite material, and back echo represents the reflection of ultrasonic waves on the lower surface of the composite material. The parameter vector of front echo is set to $\theta_{F}=\left[40,\;0.5\;\mu s,\;3.5\;\mathrm{MHz},\;2.5,\;1\right]$, the parameter vector of back echo is set to $\theta_{B}=\left[50,\;2\;\mu s,\;3.3\;\mathrm{MHz},\;1,\;0.3\right]$. It is important to note that compared with ordinary sound, the frequency of ultrasound is higher, and ultrasound attenuates more quickly in the air. The delamination defects of carbon fiber laminates generally contain air, and ultrasound cannot penetrate the delamination defects to reach the lower surface. As a result, the A-scan signal of the delamination area obtained by the ultrasonic transducer generally has no back echo. Therefore, the A-scan signal of defect area can be generated by adding only front echo and defect echo, as shown in Figure 3b,c, where their front echo is the same as that of Figure 3a, the defect echo represents the reflection of ultrasonic waves on the delamination defect. The parameter vector of defect echo 1 is set to $\theta_{1}=\left[60,\;1\;\mu s,\;3.3\;\mathrm{MHz},\;2,\;0.5\right]$ and the parameter vector of defect echo 2 is set to $\theta_{2}=\left[60,\;1.5\;\mu s,\;3.3\;\mathrm{MHz},1.5,\;0.4\right]$.

**Figure 5:**
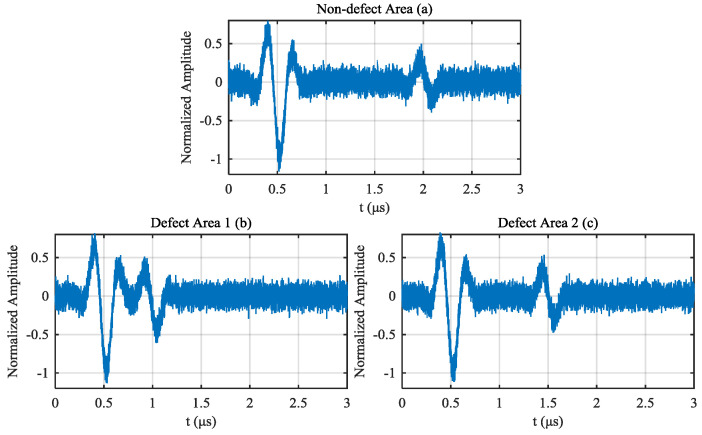
Noisy signal generated from (**a**) non-defect area, (**b**) defect area 1 and (**c**) defect area 2.

White noise with an SNR of 25 dB is added to those A-scan signals. For the A-scan signal of non-defect area, 10 noisy signals are generated, five of which are used to generate the reference signal, and the other five signals are used to compare with signals of defect area. For the A-scan signals of defect area 1 and defect area 2, five noisy signals are generated for comparison, respectively. Figure 5 shows one of noisy signal generated from signal of non-defect area, signal of defect area 1 and signal of defect area 2, respectively.

**Figure 6:**
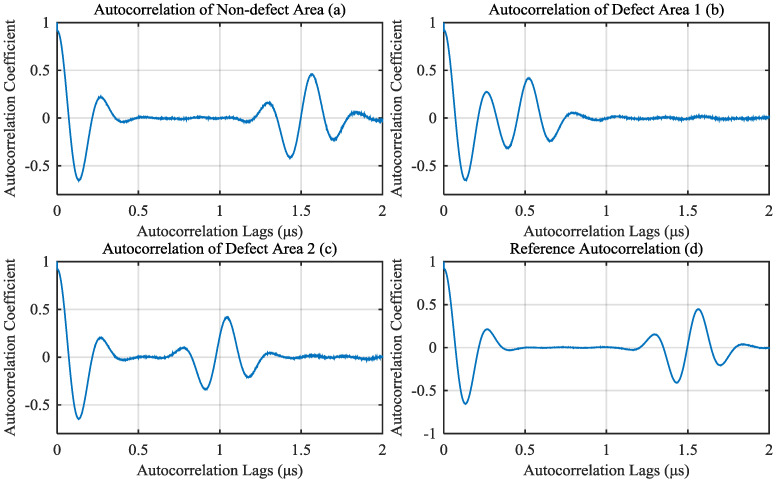
Autocorrelation of (**a**) non-defect signal; (**b**) defect 1 signal and (**c**) defect 2 signal; (**d**) calculated reference autocorrelation signal.

Calculate the autocorrelation of those noisy signals. Because the thickness of the object to be test is 0.75 μs sound path, the time between front echo and back echo is 1.5 μs. So the region of interest (autocorrelation lags from 0 to 2 μs) can be intercepted from autocorrelation. The correlation results calculated from signals of Figure 5 are shown as Figure 6a–c. Calculate the reference autocorrelation signal from A-scan signals of non-defect area by the algorithm proposed in Section 2.4, as shown in Figure 6d.

The Euclidean distances between the reference autocorrelation and autocorrelation of five signals which are used to generate reference autocorrelation are 0.4068, 0.2988, 0.3524, 0.3827, 0.3935, respectively. As a result, three times the average value (0.3668) is calculated as the threshold value (1.1005). Calculate the Euclidean distance between the reference autocorrelation and autocorrelation of 15 signals from non-defect area and two kinds of defect area to be classified, respectively. The results are shown in Table 1. It can be found that non-defect signals and defect signals can be classified well according to the threshold value, 1.1005.

**Figure 7:**
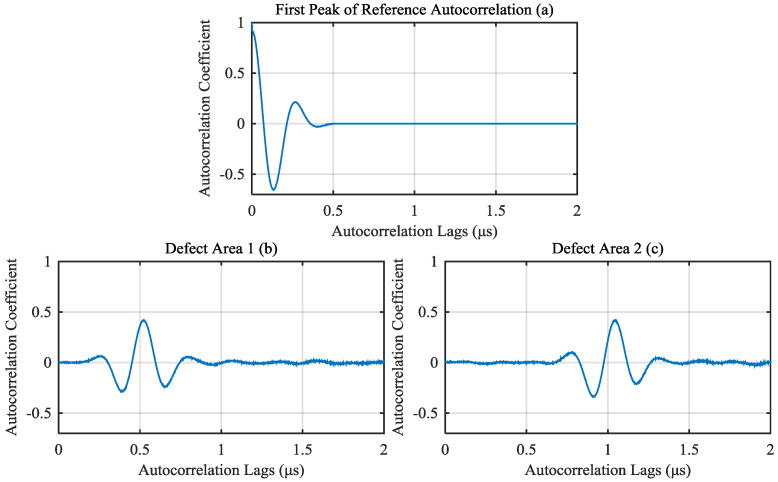
(**a**) First peak of the reference autocorrelation. Autocorrelation (after subtracting the first peak) of signals from (**b**) defect area 1 and (**c**) defect area 2.

For A-scan signals of defect area, calculate the depth of the defect by the correlation method proposed in this paper. First, we should eliminate the impact of the first peak of autocorrelation, which can be intercepted from reference autocorrelation. In this simulation, from 0 to 0.5 μs autocorrelation lags of reference autocorrelation is intercepted as the first peak, as shown in Figure 7a. The first peak is subtracted from autocorrelation of defect signals. The results of defect signals in Figure 6b,c are shown in Figure 7b,c, respectively.

Finally, calculate the defect depth from the autocorrelation after cut first peak. The result is shown in Table 2. It can be found that using the algorithm proposed in this paper, the error in calculating the depth of the defect is within 3.5%, and the error is larger when the depth is near the top surface. The reason for this result is cross-aliasing of defect echo and front echo, which leads to difficulties in separating the defect echo from the front echo. Therefore, the depth calculation of near-surface defect is not as accurate as that of non-near-surface defect.

## 4. Experimental Test Result And Discussion

**Figure 8:**
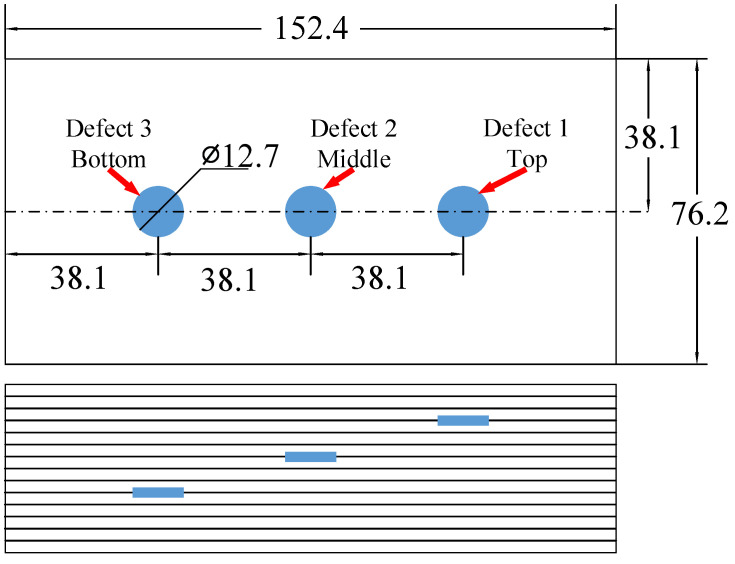
Schematic diagram of carbon fiber laminate (total 14 layers). Three graphite disks were inserted between 3rd and 4th layers, 6th and 7th layers and 9th and 10th layers, respectively, as the artificial delamination defects. (unit: mm).

The experiment was conducted in a test carbon fiber laminate with three circular delamination defects in different depth. The laminate was 14 layers and in total 2 mm thick and made by autoclave. The laminate sample layering method was ${\left[90/0\right]}_{7}$, where the $0^{\circ }$ direction was the long side direction. Three artificial delamination defects were simulated by inserted graphite disks. All these graphite disks were round and the same size, with a diameter of 12.7 mm. Figure 8 shows a schematic diagram of the carbon fiber laminate. The three graphite disks were located between 3rd and 4th layers, 6th and 7th layers and 9th and 10th layers, respectively. These three graphite disks have the same thickness, 0.25 mm. During the forming process of autoclave, the shape, size and depth of artificial defects inevitably change slightly. Therefore, the actual defect size and location were no longer the same as the expected. The detection result obtained by phased array ultrasonic testing equipment was used as a standard of artificial defects. The comparing results are elaborated in Section 4.4 (defect depth) and Section 4.5 (shape, size of defects and imaging result).

**Figure 9:**
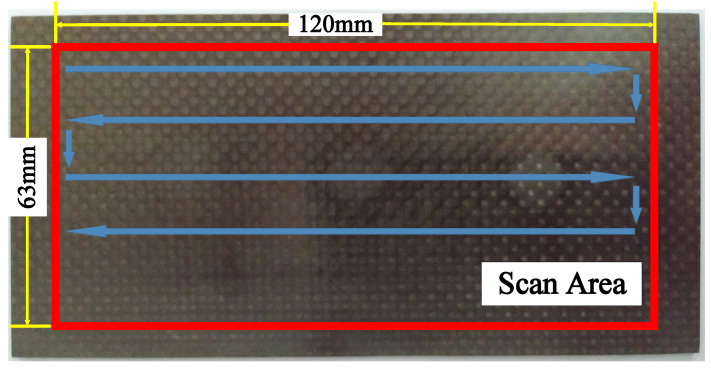
Scan area and path.

**Figure 10:**
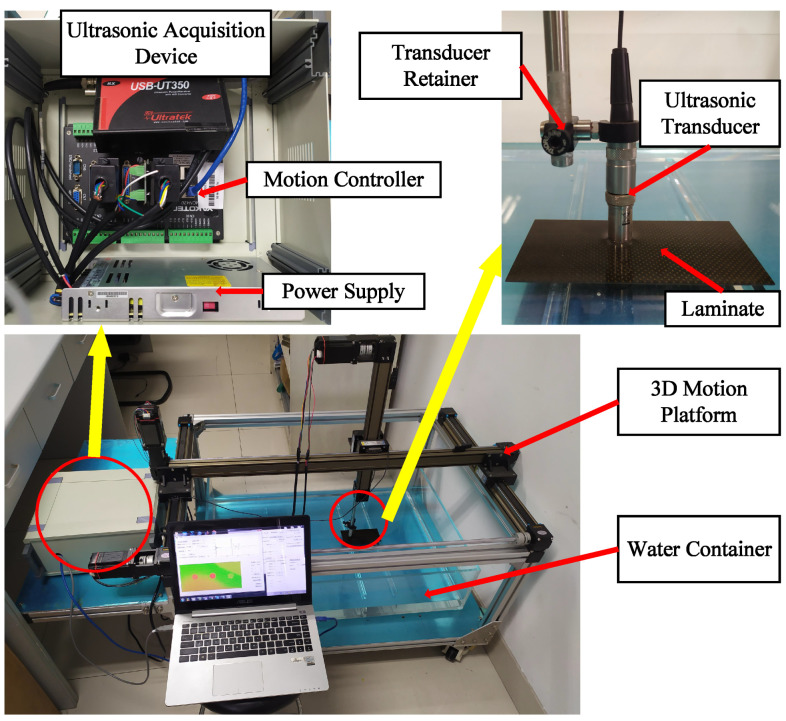
Ultrasonic immersion scanning system.

A ultrasonic water immersion scanning system was utilized to get A-scan signals, in which a 5 MHz ultrasonic transducer (V310-SU-F0.50IN-PTF, Olympus, Tokyo, Japan) and a ultrasonic acquisition device (USB-UT350, US Ultratek, Walnut Creek, CA, USA) were used. The scan area and path are shown in Figure 9. The ultrasonic excitation pulse voltage was set to 40 V and width 50 ns. The ultrasonic acquisition sampling rate was set to 100 MSPS (mega samples per second). The system is shown in Figure 10. The immersion ultrasonic inspection was conducted in pulse/echo model. The scanning interval was 0.375 mm. The distance between the ultrasonic transducer and the laminate was set to 7.5 mm.

**Figure 11:**
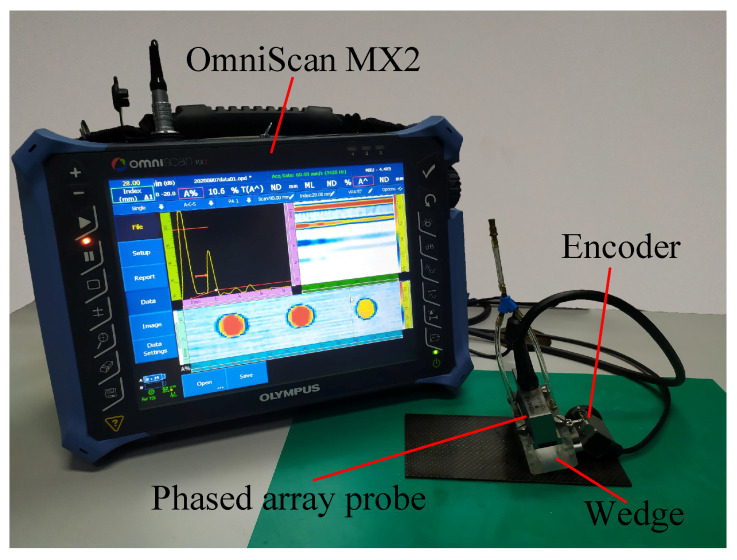
Phased array ultrasonic testing system.

The phased array ultrasonic testing equipment for comparison is shown in Figure 11. The equipment model was Olympus OmniScan MX2. A phased linear array probe (5L64-NW1, Olympus, Tokyo, Japan) with 64 chips, 1 mm chip center spacing and 5 MHz center frequency was selected. To overcome the blind zone near the surface of the probe, we selected a 22 mm high plexiglass wedge (SNW1-0L, Olympus, Tokyo, Japan). Laminates and wedges were coupled with pure water. We obtained the scanning position through the encoder (ENC1-2.5-DE, Olympus, Tokyo, Japan). Both ultrasonic water immersion scanning and phased array scanning experiments were carried out indoors.

### 4.1. Signal Filtering

**Figure 12:**
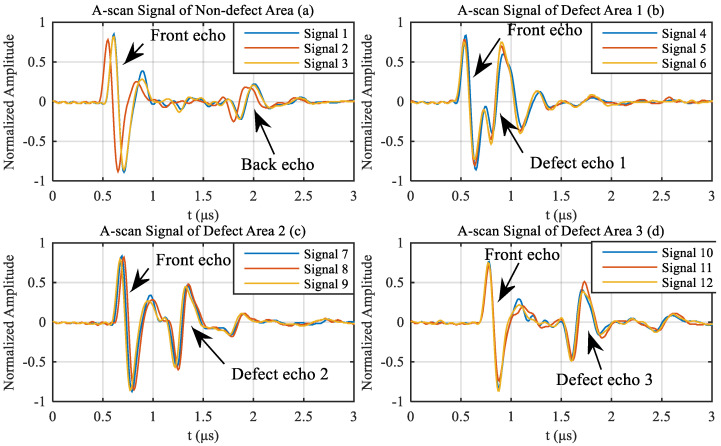
Representative A-scan signals from (**a**) non-defect area and three artificial delamination defect areas: (**b**) defect 1 (top defect); (**c**) defect 2 (middle defect); (**d**) defect 3 (bottom defect).

Because of the 5 MHz ultrasonic transducer, band pass filter from 3 MHz to 8 MHz was used to reduce the noise in A-scan signals. The Figure 12 shows four condition (from non-defect area (a) and three delamination defects in three different depth (b), (c) and (d)), of each displays three representative A-scan signals.

Because ultrasound can not penetrate delamination defect, we could not find back echo in A-scan signals of defect area, as shown in Figure 12b–d. Due to the attenuation of ultrasound in the carbon fiber laminate, it was arranged in descending order of amplitude as front echo, defect echo 1, defect echo 2, defect echo 3, back echo. We needed to add TCG (time compensation gain) when testing more thicker carbon fiber laminates, especially thickness greater than 15 mm, to make back echo visible. TCG makes the deeper echo have greater gain. In this experimental test, we did not add TCG because of the 2 mm thickness laminate and visible back echo.

### 4.2. Generating Reference Signal

**Figure 13:**
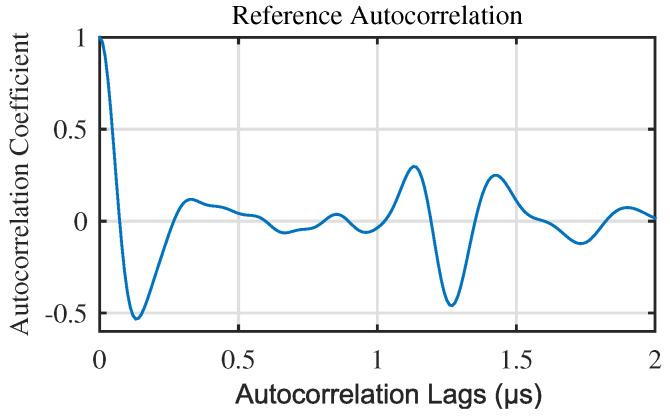
Result of calculated reference autocorrelation.

The reference signal was calculated from non-defect A-scan signals by the proposed correlation method. A total of 10 A-scan signals from different non-defect positions were collected for this step. The result is shown in Figure 13.

### 4.3. Signal Classification And Results

Euclidean distance was used to classify defect and non-defect signals. The calculate results are shown in Table 3. For the collected 10 signals from non-defect area which was used to calculate the reference correlation, the minimum Euclidean distance was 0.1855, the maximum Euclidean distance was 0.5050, average was 0.3406. According to the method proposed in this paper, the threshold was set as 1.0218. For each defect in different depth, 10 signals were collected, typically. Their minimum Euclidean distance were 2.2639, 2.4829, 2.4303, maximum Euclidean distances were 2.5701, 2.6179 and 2.6140, averages were 2.4115, 2.5786 and 2.5222. From Table 3, it can be found that non-defect signals and defect signals could be well classified according to the threshold value 1.0218.

### 4.4. Defect Depth Calculation And Comparing

**Figure 14:**
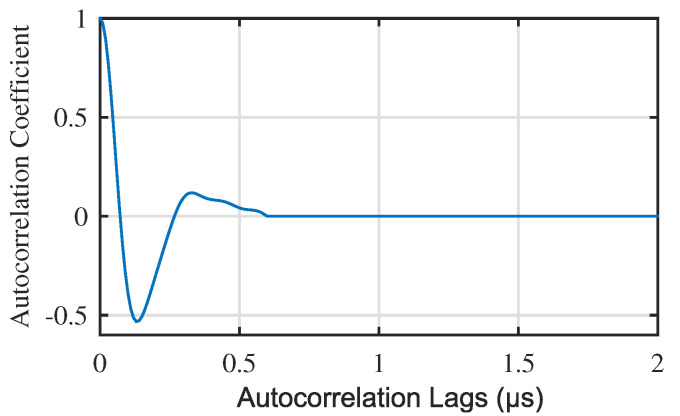
First peak of reference autocorrelation.

For defect signals obtained by classification, the defect depth calculating was essential. The first peak of correlation should be cut because of its impact of depth calculation. In this simulation, from 0 to 0.6 μs autocorrelation lags of reference autocorrelation was intercepted as the first peak, as shown in Figure 14.

**Figure 15:**
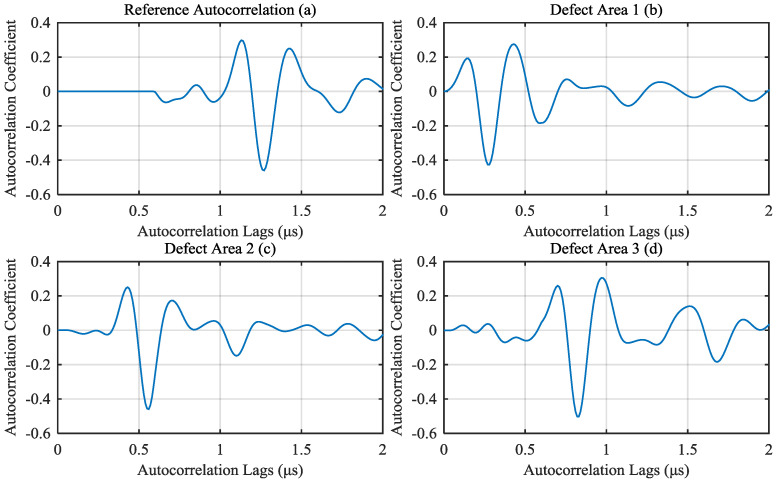
Results of subtracting the first peak from (**a**) reference autocorrelation and autocorrelation of (**b**) Defect Area 1 A-scan signal; (**c**) Defect Area 2 A-scan signal; (**d**) Defect Area 3 A-scan signal. (The A-scan signals are shown in Figure 12b,c,d, respectively).

The first peak was subtracted from autocorrelation of defect signals. The results of defect signals from three different defect area in Figure 12b–d are shown in Figure 15b–d, respectively. Figure 15a is the reference autocorrelation after cut first peak.

**Figure 16:**
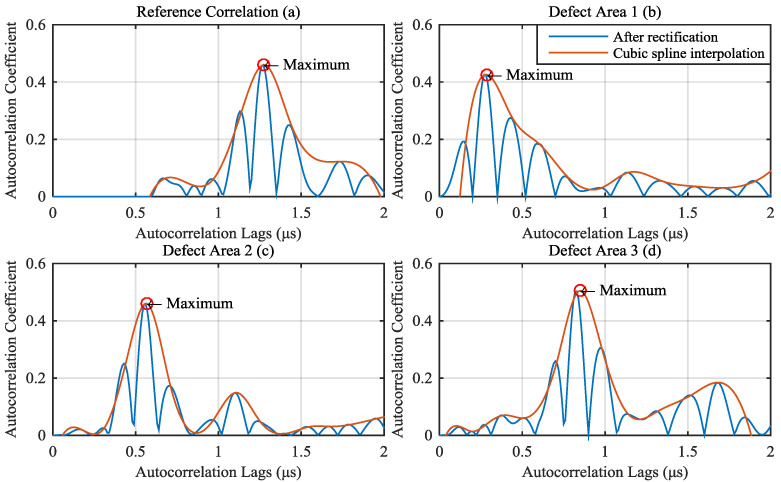
Results of full wave rectified (the blue line) and cubic spline interpolation (the red line). (**a**) reference; (**b**) defect area 1; (**c**) defect area 2 and (**d**) defect area 3 correspond to Figure 15a–d.

To facilitate the following calculation, the full wave rectified signals were calculated from defect autocorrelation (after cut first peak) which was classified in Section 4.3. The cubic spline interpolation was used to calculated the time-of-flight (TOF). The result is shown in Figure 16, in which (a), (b), (c) and (d) were calculated from Figure 15a–d, respectively. From the result in Figure 16a and the thickness of the carbon fiber laminate (2 mm), the sound speed in the laminate can be calculated. It can be calculated from Figure 16a that the double sound path was 1.277 μs and the sound path was 0.6385 μs. Therefore, the sound speed in the carbon fiber laminate was 3132.3 m/s.

The depths of defects 1, 2 and 3 obtained from Olympus OmniScan MX2 were 0.41 mm, 0.86 mm, and 1.32 mm, respectively, as a standard for verifying the results of the proposed algorithm for calculating the depth of defects. The defect depth was calculated from 30 defect signals collected in Section 4.3, as shown in Table 4. It can be found from the table that using the algorithm proposed in this paper, the error of all sample in calculating the depth of defect 2 and 3 was less than 3%. Although the variance of the calculation result of the defect depth was larger than that of defect 2 and 3, the average error of the calculated depth was still less than 3%. The reason is because of the cross-aliasing of defect echo and front echo, which led to inaccurate separation of the defect echo and the front echo.

Table 5 shows the comparison of defect depth from conventional ultrasonic C-scan and proposed algorithm. It can be found from the table that the proposed algorithm can measure the defect depth more accurate than conventional ultrasonic C-scan method, especially when measuring near surface defect.

### 4.5. Defect Imaging And Comparing

**Figure 17:**
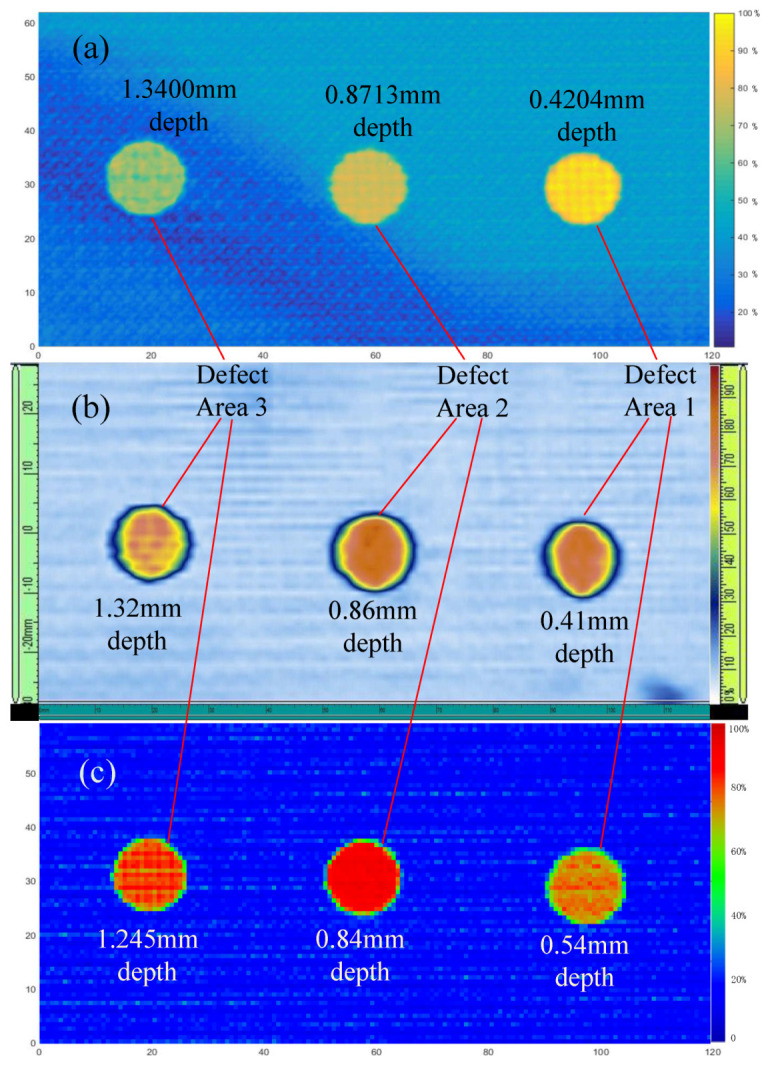
Defect imaging result of (**a**) the proposed algorithm, (**b**) ultrasound phased array C-scan and (**c**) conventional ultrasonic C-scan. (unit: mm).

The scan result was made into a 2D image, which was similar to ultrasonic C-scan image, as shown bellow. The Figure 17a is the result of the proposed algorithm. Scales of the colorbar on the right side of the Figure 17a represents the normalized Euclidean distance. In Figure 17a, the yellower the color, the greater the Euclidean distance between the reference autocorrelation and the autocorrelation of the ultrasound A-scan signal at its location. The reference autocorrelation is calculated from the echo signal of the defect-free position, so the yellower the color, the more different the ultrasonic echo signal of the position from the ultrasonic echo signal of the defect-free position. In other words, the larger the defect here. The Figure 17b is the result from Ultrasound phased array C-scan method, which was obtained by Olympus OmniScan MX2. Scales of the colorbar on the right side of the Figure 17b represents the amplitude of the defect echo. In the Figure 17b, the redder the color, the higher the echo amplitude of the defect, and the larger the defect here. The Figure 17c is the result from conventional ultrasonic C-scan method. As with Figure 17b, the redder the color, the higher the echo amplitude of the defect, and the larger the defect here.

From left to right, defect area 3, 2 and 1, respectively. In order to better distinguish the three defect areas, the defect depth calculated by the proposed algorithm is marked on Figure 17a, the standard defect depth measured by the phased array ultrasonic equipment is marked on Figure 17b, and the defect depth obtained by conventional ultrasonic C-scan method is marked on Figure 17c. Because of the inevitably slight change of shape, size and depth of artificial defects, the actual defect size and location weere no longer the same as the Figure 8 carbon fiber laminate drawing. Therefore, the result of Olympus OmniScan MX2 (as shown in Table 6) is also used as a standard result to verify the accuracy of the results of the proposed algorithm. Meanwhile, the defect size was also obtained by conventional ultrasonic C-scan method for comparison. The size calculation result and error of these three defects are shown in Table 6.

From the Figure 17a,b, it can be found that compared with the ultrasound phased array C-scan, the proposed algorithm can show the shape the defect well. Because of the 0.375 mm scanning interval, the defect size (width and length) cannot be more accurate, especially for defect 1 (near-surface defect). However, the error of both width and length calculated by the proposed algorithm is less than 4%.

From Table 6, we can find that both the proposed algorithm and conventional ultrasonic C-scan method can calculate the defect depth well. However, compared with conventional ultrasonic C-scan method, the proposed algorithm can calculate the near upper surface defect more accurate.

### 4.6. Comparison of Phased Array C-Scan, Conventional Ultrasonic C-Scan and Proposed Algorithm

Through experimental test and the calculation result of defect depth and size (Section 4.4 and Section 4.5), we get the following comparison result.

(1) The phased array ultrasonic C-scan has higher detection accuracy and speed than conventional ultrasonic C-scan and the proposed algorithm.
(2) The proposed algorithm can avoid signal peak tracking and complex gate setting, which are necessary when using phased array ultrasonic C-scan and conventional ultrasonic C-scan. The proposed algorithm needs less prior knowledge, more convenient for operators to measure objects and more suitable for automated testing.
(3) Compared with conventional ultrasonic C-scan method, the proposed algorithm can calculated depth and size of the near surface defect better.
(4) In addition, we found in actual measurement that the proposed algorithm is more sensitive to the surface of the object. As a result, when the surface of the object to be tested is uniform, the proposed algorithm performs better.

## 5. Conclusions

The experiments in this paper show that through the comparison with the result obtained by Olympus OmniScan MX2, the proposed correlation algorithm performs well in the defect shape presentation and the calculation of depth and size. Without prior knowledge of echoes, especially without the complicated gate setting of traditional ultrasound C-scan, the proposed method can generate defect images similar to C-scan based on signal classification and correlation.

The advantages of the algorithm proposed in this paper are as follows.

(1) By using signal autocorrelation instead of the original ultrasonic pulse-echo signal, some problems can be avoided, such as complex gate setting and signal peak tracking because of the slight change in the distance between the ultrasonic transducer and the laminate which can lead to signal peak time shift.
(2) The proposed algorithm only requires a small amount of reference signals in non-defect areas, without prior knowledge and adjustment of parameters such as gates and thresholds.
(3) The proposed algorithm can detect the depth and size of defects with high precision. The defect size error is less than 4%, and the defect depth error is less than 3%. Provides a high-precision ultrasonic detection and signal processing method.
(4) The proposed algorithm provides a new idea and direction for ultrasonic visual testing and can be widely used in automated ultrasonic testing.

## Figures and Tables

**Table 1:** Euclidean distance between the reference and autocorrelation to be classified.

Sample	Non-Defect Area	Defect Area 1	Defect Area 2
1	0.3733	7.1439	7.1929
2	0.4343	7.1246	7.3927
3	0.5421	7.1526	7.4258
4	0.4091	7.3631	7.3605
5	0.4995	7.1424	7.3247

**Table 2:** Results of defect depth (unit: 1 μs sound path).

Sample	Defect Area 1 (Depth 0.25)		Defect Area 2 (Depth 0.5)	
	Depth	Error (%)	Depth	Error (%)
1	0.2568	2.72	0.5037	0.74
2	0.2579	3.14	0.5087	1.73
3	0.2573	2.92	0.5104	2.08
4	0.2570	2.80	0.5085	1.70
5	0.2572	2.88	0.5032	0.64

**Table 3:** Results of calculated Euclidean distance.

Sample	Non-Defect Area	Defect Area 1	Defect Area 2	Defect Area 3
1	0.3337	2.5523	2.5838	2.5079
2	0.3790	2.5026	2.5202	2.4704
3	0.2483	2.3818	2.4829	2.4303
4	0.2769	2.2894	2.6116	2.5084
5	0.3649	2.2639	2.6179	2.5416
6	0.3048	2.2975	2.6149	2.5653
7	0.1855	2.3653	2.5982	2.5945
8	0.4699	2.4036	2.5936	2.4551
9	0.5050	2.4889	2.5563	2.5349
10	0.3381	2.5701	2.6070	2.6140

**Table 4:** Calculated result and error of defect depth.

Sample	Defect Area 1 (0.41 mm Depth)		Defect Area 2 (0.86 mm Depth)		Defect Area 3 (1.32 mm Depth)	
	Depth (mm)	Error (%)	Depth (mm)	Error (%)	Depth (mm)	Error (%)
1	0.4401	7.3400	0.8833	2.7117	1.3312	0.8519
2	0.4401	7.3400	0.8802	2.3474	1.3359	1.2078
3	0.4291	4.6660	0.8708	1.2548	1.3422	1.6824
4	0.4072	0.6819	0.8661	0.7084	1.3438	1.8011
5	0.3962	3.3558	0.8661	0.7084	1.3485	2.1570
6	0.4150	1.2281	0.8677	0.8905	1.3438	1.8011
7	0.4197	2.3741	0.8692	1.0726	1.3281	0.6146
8	0.4276	4.2840	0.8708	1.2548	1.3375	1.3265
9	0.4150	1.2281	0.8739	1.6190	1.3406	1.5638
10	0.4135	0.8461	0.8645	0.5263	1.3485	2.1570
Average	0.4204	2.5269	0.8713	1.3094	1.3400	1.5163

**Table 5:** Comparison of defect depth from conventional ultrasonic C-scan and proposed algorithm

Measurement Method		Defect Area 1	Defect Area 2	Defect Area 3
Olympus OmniScan MX2 (mm)		0.41	0.86	1.32
Conventional ultrasonic C-scan	Depth (mm)	0.54	0.84	1.245
	Error (%)	31.707	2.3256	5.6818
Proposed algorithm	Depth (mm)	0.4204	0.8713	1.3400
	Error (%)	2.5269	1.3094	1.5163

**Table 6:** Results of calculated defect size. (mm)

Detect Items		OmniScan MX2	Proposed Algorithm	Error	Conventional C-Scan	Error
Defect Area 3	Length	12.90	13.125	1.744%	13.5	4.444%
	Width	12.55	12.75	1.594%	12.75	1.594%
Defect Area 2	Length	13.00	12.75	1.923%	13.125	1.154%
	Width	12.95	13.125	1.351%	13.125	1.351%
Defect Area 1	Length	11.90	12.375	3.992%	12.75	7.143%
	Width	12.35	12.75	3.239%	12.75	6.275%
